# The emerging role of RNAs in DNA damage repair

**DOI:** 10.1038/cdd.2017.16

**Published:** 2017-02-24

**Authors:** Ben R Hawley, Wei-Ting Lu, Ania Wilczynska, Martin Bushell

**Affiliations:** 1 MRC Toxicology Unit, Leicester, UK

## Abstract

Many surveillance and repair mechanisms exist to maintain the integrity of our genome. All of the pathways described to date are controlled exclusively by proteins, which through their enzymatic activities identify breaks, propagate the damage signal, recruit further protein factors and ultimately resolve the break with little to no loss of genetic information. RNA is known to have an integral role in many cellular pathways, but, until very recently, was not considered to take part in the DNA repair process. Several reports demonstrated a conserved critical role for RNA-processing enzymes and RNA molecules in DNA repair, but the biogenesis of these damage-related RNAs and their mechanisms of action remain unknown. We will explore how these new findings challenge the idea of proteins being the sole participants in the response to DNA damage and reveal a new and exciting aspect of both DNA repair and RNA biology.

## Facts

- The miRNA biogenesis machinery has a role in DNA damage repair outside of canonical miRNA-mediated translational repression.
- RNA molecules have been observed in the proximity of DNA breaks and have been implicated in the DNA repair response.
- These phenomena have been observed in many species, indicating an evolutionarily conserved mechanism.

## Open Questions

- What is the precise role of the RNA-processing enzymes in DNA repair?
- Do small RNAs have a direct mechanistic role in DNA repair, or do they serve as a by-product of a different RNA species?
- Is transcription induced locally at sites of DNA damage? Are proximal dormant promoter elements involved, or is an open-ended break sufficient for polymerase recruitment?
- Can these results be replicated outside of integrated exogenous reporter systems?

## An Unlikely Match: RNA Biogenesis Machinery Meets DNA Repair

Our DNA is constantly exposed to various environmental and chemical agents, including ionising radiation (IR) from cosmic radiation, ultraviolet (UV) light from the sun or even nucleophilic attack induced by chemical compounds in food.^1^ In fact, DNA damage is intrinsic to the process of life: it is inevitable during replication and essential during meiotic recombination. Also, controlled DNA breaks by topoisomerase occur to facilitate the resolution of supercoiled chromatin structures. Complex mechanisms have evolved to counteract the variety and quantity of DNA damage encountered daily.

**Figure 1:**
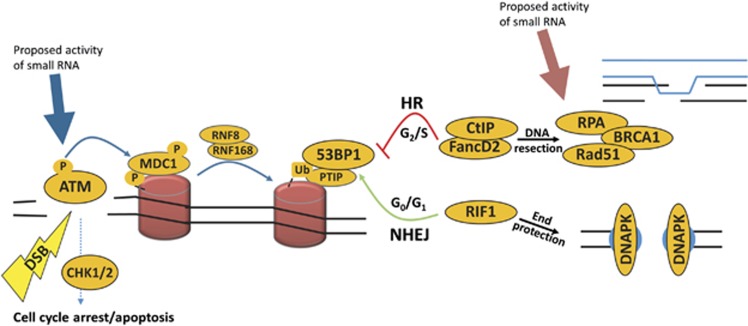
A schematic of the DNA repair pathway. The formation of a DSB induces the phosphorylation of ATM, which contributes to the activation of the DNA repair pathway and cell cycle arrest. A series of molecular signalling events lead to the deployment of ubiquitylation (Ub) marks on the histones (red cylinders) in the proximity of DNA breaks, facilitated by RNF8 and RNF168. The recruitment of 53BP1 marks the key crossroad of DSB repair (DSBR) pathway, which branches out into error-free HR or relatively error-prone NHEJ. Small RNAs have been proposed to function at two distinct steps in DSBR. Francia *et al.*^11^ suggested that it affects early signal propagation through ATM phosphorylation (blue arrow), while Gao *et al.*^8^ proposed that it only affects the HR sub-pathway via modulation of Rad51 binding (red arrow)

Generally, DNA damage response (DDR) involves a complex signalling cascade initiated by one of three PI3K-like kinases: ATM, ATR or DNA-PK. They serve to facilitate chromatin modification and remodelling, allowing access to and acting as scaffolds for proteins involved in repair, as well as propagating the damage signal.^1^ Many of these recruited factors are involved in a binary decision-making process (see Figure 1). The repair of double-strand breaks (DSBs) is resolved by two distinct mechanisms: error-free homologous recombination (HR) or error-prone non-homologous end-joining (NHEJ).^1, 2^ The choice of which mechanism is used can depend on chromosomal context and is cell cycle stage dependent;^3^ HR is favoured when sister chromatids are available in G2 phase, whereas NHEJ is favoured over HR in the G1 stage of the cell cycle, and in resting or terminally differentiated cells.^2, 4^ Commitment to the HR pathway is facilitated by the eviction of key repair proteins, such as 53BP1, from the damage site.^2^ This is followed by the recruitment of pro-HR proteins, such as BRCA1, FancD2 and CtIP, leading to the resection of DNA around break sites and the search of homologous chromatids for template-mediated repair.^5^ Conversely, stabilisation of 53BP1 at break sites by PTIP and Rif1 blocks resection, causing NHEJ to occur.^6, 7^

**Figure 2:**
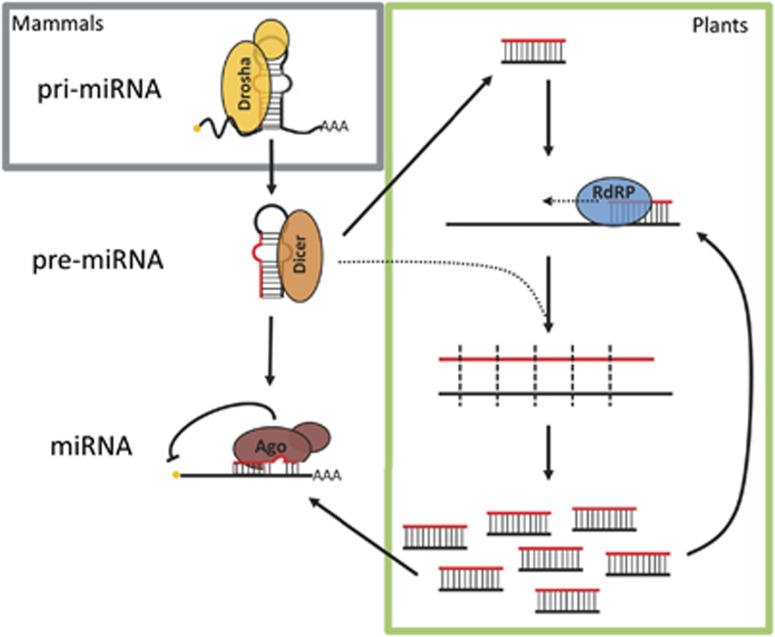
Outline of the microRNA biogenesis pathway in humans, and how plants utilise RdRPs to amplify these. The miRNA gene is transcribed by RNA polymerase II and typically capped and polyadenylated. This primary miRNA (pri-miRNA) contains the hairpin structure that is recognised and cleaved by Drosha, as part of the Microprocessor complex. The stem loop is then further trimmed by Dicer forming the pre-miRNA. In the canonical miRNA pathway, a single strand of the small RNA duplex is loaded into an Argonaute protein (Ago), which leads to repression of target transcripts. In plants, a dsRNA precursor is cleaved by Dicer into small dsRNA (green box). There exist multiple amplification pathways; broadly, an RdRP can synthesise a complementary strand by elongating a small RNA bound to its target RNA. Plant Dicer proteins can then cleave this newly generated dsRNA. to produce many secondary siRNAs that can repress target transcripts via Ago, or begin another cycle of small RNA amplification

Traditionally, it has been thought that DNA repair involves only enzymatic reactions carried out by proteins that facilitate repair and propagate signalling events. Interestingly, a number of reports have now implicated RNA in DDR.^8, 9, 10^ These have largely concentrated on the involvement of the small RNA biogenesis enzymes (outlined in Figure 2) and have identified a novel species of small RNA, which appears to be derived from the vicinity of the DSB. The involvement of an RNA species in DDR is well-conserved evolutionarily with observations in fungi, yeast, plant, *Drosophila* and human cells.^9, 11, 12, 13, 14, 15^ The first description came from the filamentous fungus *N. crassa,* where interplay between non-canonical small RNAs and the DDR was reported. Chemically induced replication stresses in *N. crassa* resulted in the production of small RNAs originating mostly from highly transcribed and repetitive ribosomal loci. This event was dependent on the presence of the fungal orthologue of Argonaute protein and an RNA-dependent RNA polymerase.^14^ Although required for proficient DNA repair, these small RNAs appeared to be produced from the degradation of longer RNA species.^14^ The authors proposed that aberrant transcripts ('aRNA') transcribed as a result of DNA damage are amplified by RNA-dependent RNA polymerases (RdRPs) and processed into small RNA (termed quelling-induced RNA, qiRNA). These qiRNAs then act to degrade aRNA, in a manner similar to the siRNA amplification cycle.^12, 16, 17^ These aRNAs are transcribed from repetitive loci, such as the ribosomal DNA locus, and are refractory to RNA polymerase inhibitors.^14^ Interestingly, the production of aRNAs is dependent on the presence of replicating protein A, a known component of the HR repair pathway.^18^ How such a mechanism could aid in repair of a break itself is unclear. Nevertheless, one can imagine the suppression of these aberrant transcripts by qiRNA serves to complement nonsense-mediated decay to limit any possible translation of abnormal transcripts.

Similarly, production of small RNAs was observed post-DNA damage in plants. The plant orthologs of Dicer protein, DCLs, are required for efficient DSB repair when *A. thalina* is challenged with IR.^9^ Utilising next-generation sequencing (NGS), it was shown that DNA damage-induced small RNAs (diRNAs) arose in the proximity of the DSB sites. Interestingly, although these diRNAs are required for proficient repair, they are not involved in the initial recognition of DSBs indicated by the continued phosphorylation of Histone H2A.X.^9^

Recently, the importance of diRNAs in the DDR pathway was highlighted in metazoa. Several publications have documented the requirement for small RNAs, or certain components of the small RNA biogenesis machinery, in proficient DNA repair signalling.^8, 10, 11, 13, 19^ It is largely agreed that the key RNAse III family enzymes that process small RNA precursors, Drosha and Dicer, have a role in the DNA repair response.^9, 10, 11^ Indeed, the loss of diRNAs or the small RNA biogenesis machinery, appears to affect the DNA repair process and have an impact on repair pathway choice.^8, 10, 16^ Which stage within the DDR pathway is affected by the loss of diRNAs and related proteins is currently contested (see Figure 1). Nevertheless, similarly to plants, it is thought that the initial phosphorylation of histone variant H2A.X is not affected by the loss of diRNAs.^8, 11^ It is also noteworthy that RNA polymerase II activity has been implicated in this process.^11^

The generation of small RNA is a multistep biological process (Figure 2). Various accessory proteins such as DGCR8 are required alongside Dicer and Drosha,^20^ but their involvement in DNA repair has not been investigated in depth. Moreover, the participation of key downstream effectors in the RNAi pathway, namely the Argonautes, is also contested.^8, 10, 11, 21^ Currently, the mechanism by which the diRNAs directly influence repair outcome is under extensive investigation. Nevertheless, it has been reported that in *Drosophila* cells, these small RNAs can serve as endo-siRNAs to suppress existing transcripts arisen from the portion of DNA harbouring the DSB, as was proposed in *N. crassa*.^13^ Thus far, the evidence supporting the existence of DNA diRNAs has come from two main sources: deep sequencing and the isolation of the small RNA fraction for use in rescue experiments, which will be discussed in detail in this review.

## Search for the One: Using NGS to Identify diRNAs

**Figure 3:**
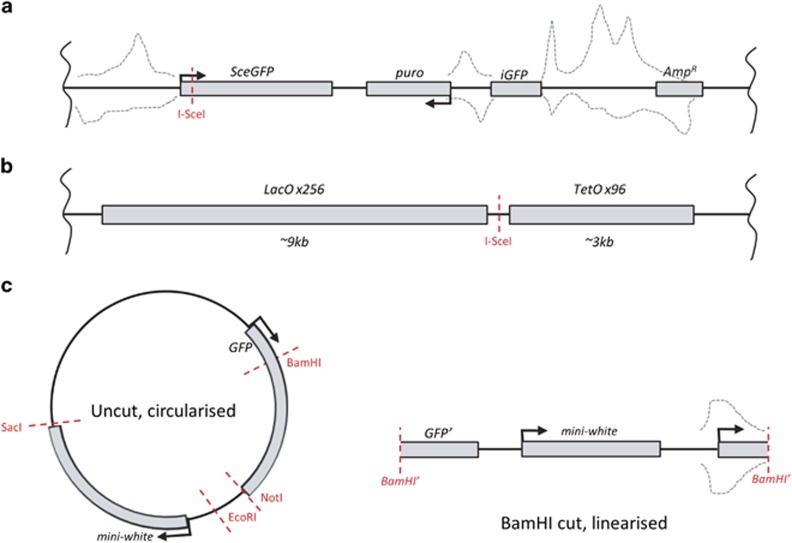
Schematics of reporter systems used for small RNA sequencing experiments. (**a**) Wei *et al.*^9^ DR-GFP genomically integrated reporter. Transfection of I-SceI results in cleavage near the transcriptional start site (TSS) of GFP. Small RNA was sequenced and mapped back to the reporter sequence. (**b**) Francia *et al.*^11^ genomically integrated Lac-/Tet-operator-flanked I-SceI site. This reporter lacks transcriptional activity but is highly repetitive. Small RNA was detected after I-SceI transfection but at low levels (47 total reads after transfection *versus* 20 reads without). No information on where these RNAs mapped to was provided. (**c**) Michalik *et al.*^13^ *Drosophila* expression plasmids, either circularised or linearised. Here only the *Bam*HI linearised vector is shown as it produced the highest number of small RNA reads. Grey dashed lines represent the approximate distribution of small RNA mapping back to the locus, where positional data was supplied in the manuscript. Right-angled arrows represent TSS, whereas vertical wavy line denotes integration within the genome

**Figure 4:**
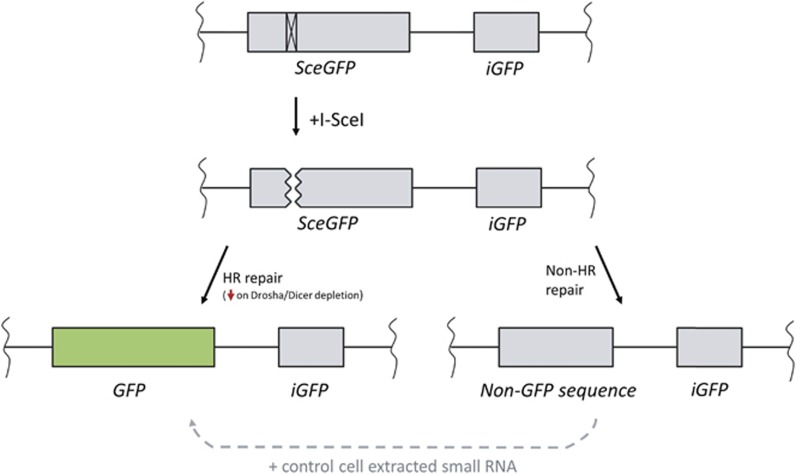
Detailed schematic of the DR-GFP HR repair reporter as in Figure 3a, used for RNA rescue experiments. A copy of the reporter is integrated into the genome to provide appropriate chromatin context. Insertion of an I-SceI restriction site within the GFP ORF results in a nonsense product that will produce no green fluorescence. After the induction of I-SceI cleavage, the cell can repair the resulting DSB via HR using the downstream internal GFP sequence (iGFP), producing a full-length GFP product. If HR is impaired, the break will instead be repaired via an alternate pathway, such as NHEJ, resulting in a sequence lacking full GFP coding region. The extent of deletion is dependent upon the non-HR mechanism chosen by the cell, but any loss of sequence within the I-SceI restriction site will prevent any further cutting. In the experiments by Wei *et al.*^9^ and Wang and Goldstein,^10^ the loss of Drosha and Dicer resulted in a lack of GFP indicating a deficiency in HR repair; however, when small RNAs extracted from control cells were incubated with these deficient cells for 1 h, GFP was found to be expressed (denoted by dashed arrow)

How can the generation of small RNA in a DNA damage-specific context be detected? Commonly used external DNA damage agents, such as IR, lead to the generation of multiple breaks at random genomic sites. This makes the task of discovery of novel RNA species with the use of an NGS approach virtually impossible, as breaks need to occur in known defined sequences for this experimental strategy to work. To date, three studies have used restriction enzyme-based systems in animal cells combined with NGS to detect diRNAs, which map to the vicinity of the cut site.^9, 11, 13^ These reports relied on the ectopic expression of rare restriction enzymes targeted to specific pre-integrated loci in the genome. Two different systems were adopted in human cell lines: the DR-GFP HR reporter or a Lac-/Tet-operator repeat-flanking reporter,^22, 23^ both of which contain a single recognition site for the uniquely cutting meganuclease I-SceI (Figures 3a and b, see also Figure 4 and the more in-depth discussion of the DR-GFP reporter assay below). Following the transfection and expression of I-SceI for 12 to 24 h, small RNAs were sequenced by NGS. These two studies reported the existence of small RNAs around the break sites.^9, 11^ However, the exact roles of these small RNAs are yet to be determined.

An alternative approach has involved the transfection of either circular (uncut) or linearised (cut) plasmids into *Drosophila* S2 cells (Figure 3c).^23^ Similarly, it was found that small RNA can be generated from the vicinity of DNA break sites. Strikingly, these small RNAs can be generated in response to either blunt or staggered DNA ends and they proceed to serve as endo-siRNAs to repress corresponding transcripts in *trans*.^13^ It is noteworthy that this response can only be provoked by a DNA DSB, but not a nicked DNA.^13^ A recent follow-up article by the same group utilised a similar GFP-based reporter to that previously described^13^ and again reported small RNAs mapping to the damaged locus following damage induction.^2, 13, 24^ In summary, these reports by Forstemann and co-workers suggest that diRNA function in *Drosophila* appears to be more similar to plant-based qiRNAs, which remove aberrant transcripts or aid in other RNA metabolic processes.^13, 14, 24^ In contrast, in mammalian cells, they are reported to have a direct contribution to the DNA repair processes.^10, 11^

Although there are many differences between the several studies published thus far, it is important to note that all the groups have found an enrichment of small RNAs mapping to exogenous loci following DNA damage. It is clear from these studies that the miRNA biogenesis enzymes and RNA species are critically involved in the DNA repair process with multiple groups presenting similar observations in a diverse collection of experimental settings. Here, we discuss these results and examine the merit of the different approaches taken, integrating them to develop new hypotheses for how RNA could participate in DNA repair.

## Needles in a Haystack: Methodologies and Difficulties of diRNA Discovery by NGS

The advent of NGS has revolutionised the RNA field, allowing robust quantitation of RNA changes between samples and discovery of novel transcripts and splice variants in a high-throughput manner. As such, NGS was the sensible choice for discovering novel RNA species at DSBs.

These small RNAs were first identified by NGS in plants and humans.^9, 11^ Reads mapping to regions proximal to the integration locus of the HR repair reporter were observed, with the earliest peaks of RNA reads visualised 12 h following the appearance of DSB induced by the transfection of I-SceI (Figure 3a).^25^ Interestingly, the bulk of these mapped RNAs do not appear to have arisen directly from the DNA break site. Instead, they were mapped to the sequence upstream of the start site of the cut GFP gene, located upstream of the puromycin resistance gene, or downstream of the homologous GFP sequence.^9^ It thus appears that the small RNAs are mapped to highly transcribed regions proximal to the break site, making it unclear as to whether they are true *de novo* transcripts, or degradation products of pre-existing mRNAs. Small RNAs were also shown to be generated in plants post-DNA damage, with read numbers considerably higher than those in human cells.^9^ One aspect of plant biology that may contribute to this difference is the existence of RdRPs (Figure 2, green box). It is possible that after DNA damage, RdRPs may be activated to amplify nascent transcripts into dsRNAs that are then further processed into diRNAs by Dicer, in a similar manner to that reported in *N. crassa*. Interestingly, the authors showed that RNA pol IV, which is responsible for transcription from repetitive and transposable elements, was critical for the production of diRNAs in plants and loss of this enzyme significantly reduced repair efficiency.^9^ In plants, RDR2 is the RdRP responsible for amplifying pol IV transcripts to produce the hc-siRNA class of small RNAs.^26, 27^ When RDR2 was ablated, the number of diRNAs was hugely reduced, however, repair efficiency was unaffected.^9^ This discrepancy between overall small RNA levels and repair efficiency suggests that the nascent RNAs produced following damage are important for repair resolution, but perhaps the amplification of secondary RNA products by RDR2 is not. This could represent a distinction in the role of small RNAs between plants and metazoa, where in animals an amplification loop is not required for a secondary role for functional RNA molecules. Alternatively, a similar mechanism may be utilising a yet undiscovered RdRP activity in animals. For example, in humans TERT-RMRP and RNA polymerase II have been demonstrated to have slight RdRP activity.^28, 29, 30^

As these reporter systems are under the control of viral promoters with high basal activity, it is entirely plausible that the pre-existing, highly abundant long RNAs transcribed from reporter loci are degraded as part of the DDR.^31^ The approach taken by Francia *et al.*^11^ partially addresses this possibility: the Tet-/Lac-flanked I-SceI sequence used is devoid of any transcriptional elements and thus any new RNAs should be generated in a DNA damage-dependent manner (see Figure 3b). Following deep sequencing, they reported a total of 47 reads arising from the 12 kb integrated locus when cut with the endonuclease, compared with 20 in uncut controls. As the parental cell lines produced no small RNAs that mapped to this sequence, these small RNAs are indeed sequence-specific and dependent on reporter integration.^11^ However, with the low read counts, and modest enrichment above background level, it is hard to convincingly conclude that new RNA species are specifically transcribed post-damage. It should also be noted that the presence of the 20 small RNA reads in the absence of damage suggests these RNAs may not be entirely damage dependent.

**Figure 5:**
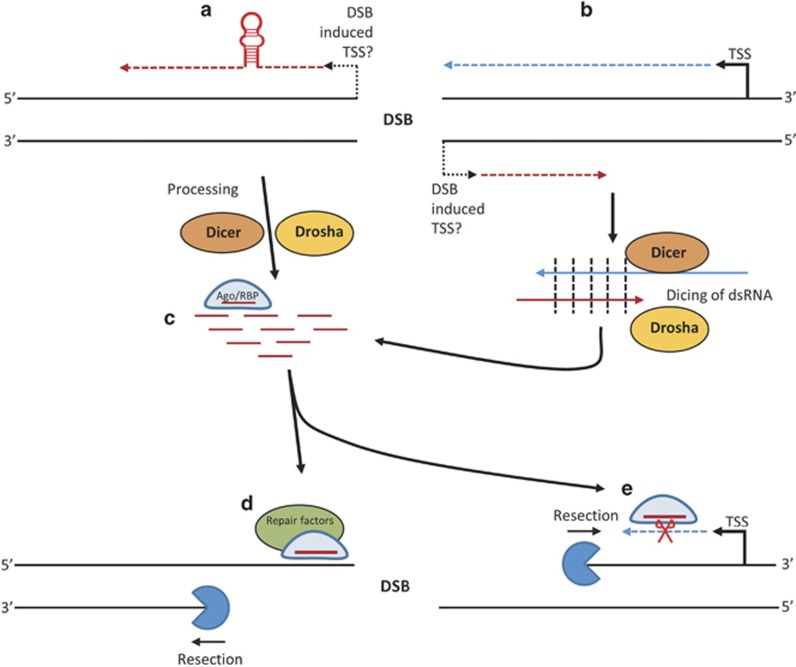
Postulated biogenesis of diRNAs and their function in DDR. Directionality of transcription, transcripts, and resection is 5' to 3', denoted by arrows. (**a**) A new transcript is formed from the break site. Any secondary structure formed by this transcript that can be recognised by Drosha and Dicer is processed into small RNAs, mirroring miRNA generation. (**b**) Where a cut occurs within an actively transcribing gene, a new end-dependent transcription event could take place resulting in formation of an antisense RNA. These RNA species can anneal to form dsRNA, and are then diced into small RNA substrates by Dicer. (**c**) The resulting small RNAs could then be incorporated into an Argonaute or another RBP, to carry out diRNA functions, which are hypothesised to be: (**d**) recruitment of repair and chromatin remodelling factors to the site of damage, (**e**) degradation of potentially aberrant transcripts or other as-of-yet unexplored functions

When similar deep sequencing investigations were performed in cells depleted of Drosha and Dicer, the analyses revealed that only loss of Dicer reduced small RNA counts significantly.^11^ The lack of a role for Drosha in production of the small RNAs but the requirement for Dicer suggests that diRNAs may be produced from the cleavage of a longer dsRNA precursor rather than from any pri-miRNA-like secondary structures within an ssRNA precursor (see also Figure 5 part a and b). It is important to remember that Drosha has been observed to impact DNA repair efficiency.^11^ This may echo the previously discussed observation in plants that generation of the small RNAs (by pol IV and RDR2) was unconnected to repair outcome. It should also be noted that Dicer and Drosha are known to have a role in non-canonical termination of new RNA transcripts.^19, 32, 33^ Whether this activity of Drosha is utilised after DNA damage warrants further investigation.

Using a comparatively straightforward system, Michalik *et al.*^13^ transfected *Drosophila* S2 cells with several exogenous sequences: a GFP expression vector and an unrelated yeast plasmid, which were either linearised ('cut') or circularised ('uncut') (see Figure 3c). A small linear PCR amplicon comprising firefly luciferase coding sequence was also used as an additional control. Following deep sequencing, small RNAs were mapped to the vector sequences with significantly more reads arising from the linearised *Drosophila* vector than the circularised one. Depending on the restriction enzyme used to generate the linearised plasmids, different patterns of small RNA were produced. This suggests the context of the DSB may affect the pattern of newly transcribed RNA. The small RNAs appeared to map predominantly upstream of the cut site, with the majority of small RNAs arising from regions adjacent to the GFP promoter. Similar to the studies conducted by Francia *et al.*^11^ and Wei *et al.*,^9^ these data do not distinguish between nonspecific degradation of RNA produced from the reporter as a result of recognition of DSB-like structures, or a deliberate processing event that generates small RNAs that may have a direct mechanistic role in DNA repair.^13^ Intriguingly, the lack of reads mapping to the PCR product, but a surprisingly high number of reads for the control yeast plasmid, suggests that some potential promoter activity may be required. One may argue that a possible pitfall of this plasmid-based approach is that the cell is exposed to DNA lacking any chromatin structure. Thus, it is hard to relate these observations to DNA damage within a genomic context. Also, it is possible that these sequences are generated in response to introduction of foreign genetic material by an anti-viral or retrotransposon defence mechanism independent of the DDR.^34, 35, 36^ However, the lack of a response from the transfected control PCR product suggests this is not the case.

Considering all these limitations, we propose that the ideal experimental setting to investigate the existence of diRNAs requires a system that produces DSBs at a range of different sites within the genome. This way, the potential involvement of chromatin structure or transcriptional status can be investigated. Thus far, two endogenous restriction enzyme-based systems have been extensively utilised in the DDR field: AsiSI^3, 37, 38^ and I-PpoI.^10, 39^ Alternatively, the CRISPR-Cas9 system also allows the induction of DSBs at specific sites of the genome:^40, 41^ using specially designed guide RNAs, the dynamics of diRNA production could be investigated even further by comparing DSBs generated proximal with promoters and transcriptional start sites to those generated further away within the same gene. Such approaches could help elucidate whether newly produced RNAs arise from transcription events at the DNA break site, or from a promoter or cryptic promoter in the vicinity of the DSBs.

## To Mend a Broken Heart: Pre-isolated Small RNA Fraction Acts in DNA Repair

The major alternative strategy used to investigate the existence of small RNAs produced following DNA damage involved the isolation of the small RNA from cells and delivery of that RNA into cells that lack the ability to produce them. Cell lines carrying a DR-GFP integrated reporter (Figure 4) were incubated with a pre-extracted small RNA fraction from damaged or undamaged cells.^9, 11^ This reporter consists of a GFP open reading frame containing an inserted I-SceI recognition site, which when transcribed results in an aberrant transcript. I-SceI induction leads to cleavage at the non-functional GFP, and allows repair via HR using the downstream intact sequence as template. This results in the creation of a copy of GFP that can express a full-length protein. The outcome of HR repair can then be measured by analysing GFP-positive cells by flow cytometry.

Depletion of Drosha or Dicer in cells carrying this transgene following 2 days of damage resulted in a reduction in GFP-positive cells indicating that HR was impaired.^9, 10^ Interestingly, two groups reported that incubation with small RNAs isolated from previously damaged cells for just 1h could restore HR efficiency.^8, 10^ In contrast, small RNAs extracted from undamaged cells failed to accomplish such rescue.^10^ This suggests the involvement of an RNA species with sequence-specific characteristics in the DDR process. The processes of transcription through to translation of a gene can take from minutes to hours, while the process of maturation and folding of fluorescent proteins may take even longer.^42, 43^ Therefore, it is unexpected that the incubation of small RNAs for merely an hour, days after the induction of DNA damage at GFP loci, can rescue expression of GFP within such a short timeframe, especially when HR repair only occurs during the S/G2-phase of the cell cycle. Moreover, given the nature of these experiments, if repair of cut sites is carried out by an error-prone non-HR mechanism, mutations will be introduced into the cut site preventing subsequent cleavage events (see Figure 4). Therefore, although it is possible for this to occur, it is unclear whether incubation with small RNAs 1 h before FACS analysis can result in restoration of functional HR repair at this specific break site.

Nevertheless, it is important to remember that this method was not the only approach used by the authors. Small RNAs were isolated from damaged cells and were able to restore 53BP1 DDR foci in cells pre-treated with RNase A.^11^

## Concluding Remarks and Future Perspectives

Recent advances in deep sequencing have made it possible to conduct refined experiments leading to the suggestion of involvement of human small RNA processing machinery in DDR. This is especially interesting given certain reports demonstrating that RNA molecules may be used as templates for DNA repair in yeast.^12^ Although it is largely agreed that Dicer and Drosha have some role in DNA repair, its mechanism is still elusive (see Figure 5).^8, 9, 10, 11^ Also, it is not clear whether this mechanism involves the typical co-factors of Drosha and Dicer, such as DGCR8, DDX5, DDX17 and TRBP. Given that Dicer and Drosha are involved in the non-canonical termination of transcription and modulation of RNA polymerase II activity, it is possible that certain interaction partners may not be required for the DNA repair-related activity.^19, 32, 33^

One primary direction for further investigation is the identity and biogenesis mechanism of the RNA species involved in DNA repair: whether these species are *bona fide* new small RNA transcripts derived from the vicinity of the break site, or degradation products of pre-existing transcripts. Whether they can serve as RNA templates (or remnants of RNA templates) that actively participate in the repair process is unknown. However, it should be noted that while several classes of non-coding RNA are produced in association with DNA damage, the impact of these RNAs on the repair process in different model systems is varied (see Table 1). For example, plant-based aRNAs, qiRNAs and hc-siRNA require RdRP activity, and they have been shown to induce the degradation of transcripts.^14, 18, 26^ Similarly, diRNAs are reported to serve as endo-siRNAs in *Droshophila* systems.^13, 24^ However, the small RNA produced in mammalian cells, termed diRNAs, are reported to be directly involved in the repair process, but the mechanism of action is still under debate.^8, 9, 10, 11^

Recent studies have provided the first direct evidence for an RNA-templated repair mechanism in both yeast and human cells, the latter of which curiously utilises the NHEJ machinery.^12, 44^ Again in yeast, an even more recent paper also demonstrates the formation of RNA:DNA hybrids at sites of DNA damage, showing a strong link between transcription and DNA repair.^45^ Alternatively, they may also be involved in the process of modulating chromatin states, in a manner similar to *piwi*-interacting RNA in germ cells.^46^ Provided that these small RNAs function in a sequence-specific manner analogous to RNAi, one should expect that an Argonaute-like protein would be required to facilitate scanning and base pairing with its genomic target (Figure 5).^47^ Currently, the jury is still out regarding the exact role of Ago2 protein in DDR.^8, 10, 21^

With recent reports documenting crosstalk between the DNA repair processes and RNA transcription, processing and splicing machinery, one can only envisage an even more intertwined interaction between RNA and the DNA repair process.^3, 25, 48^

## Figures and Tables

**Table 1:** DNA damage-specific non-coding RNA classes

**RNA class**	**Name**	**Organism**	***De novo*** **transcription**	**Requirement of RdRP**	**Direct involvement in DDR**	**Requirement of Argonaute proteins**	**Size**	**Remarks**	**Reference**
aRNA	Aberrant RNA	*N. crassa*	Yes, require QDE1	Yes, QDE-2	No	Not known	100 nt–2 kb	Serves as a precursor of qiRNA.	^14^
diRNA	DNA damage-induced RNA	*A. Thaliana*, *Drosophila*, mammalian cells	Yes, RNA polymerase II	Not known (but presumed no due to low availability of RdRP in mammalian cells)	Yes	Yes, interacts with Ago2	21–23 nt	Generated after DNA damage. Required for proficient repair of DNA damage but not for the recognition of DNA breaks.	^9, 10, 11^
hc-siRNA	Heterochromatic-associated siRNA	*A. Thaliana*	Yes, RNA polymerase IV	Yes	No	Not known	24 nt	Guide cytosine methylation at heterochromatic sites in plants.	^26, 27^
qiRNA	Quelling-induced RNA	*N. crassa*	No, generated from aRNA	Yes, QDE-2	No	Yes	20–21 nt	DNA damage induced, generated from aRNA. Strong preference of Uracil at 5'-end. Mostly originated from ribosomal DNA locus. Possibly controls rRNA biogenesis after DNA damage.	^14, 18^
No specific name given	Small RNA generated from DSB ends	*D. melanogaster*	Yes	Not known (but presumed no due to low availability of RdRP in *Droshophila*)	No	Yes	21 nt		^13, 24^
